# Epigenetic priming by Dot1l in lymphatic endothelial progenitors ensures normal lymphatic development and function

**DOI:** 10.1038/s41419-019-2201-1

**Published:** 2020-01-06

**Authors:** Hyunjin Yoo, Young Jae Lee, Chanhyeok Park, Dabin Son, Dong Yoon Choi, Ji-Hyun Park, Hee-Jin Choi, Hyun Woo La, Yun-Jung Choi, Eun-Hye Moon, Dieter Saur, Hyung Min Chung, Hyuk Song, Jeong Tae Do, Hoon Jang, Dong Ryul Lee, Chankyu Park, Ok-Hee Lee, Ssang-Goo Cho, Seok-Ho Hong, Gu Kong, Jin-Hoi Kim, Youngsok Choi, Kwonho Hong

**Affiliations:** 1 Department of Stem Cell & Regenerative Biotechnology, Humanized Pig Research Center (SRC), Konkuk University, Seoul, Gwangjin-gu 05029 Republic of Korea; 2 Lee Gil Ya Cancer and Diabetes Institute, Korea Mouse Phenotyping Center (KMPC), Gachon University, Incheon, Yeonsu-gu 21999 Republic of Korea; 3 Division of Translational Cancer Research, German Cancer Research Center (DKFZ) and German Cancer Consortium (DKTK), Baden-Württemberg, Heidelberg 69120 Germany; 4 Department of Medicine II and Institute of Translational Cancer Research, Klinikum rechts der Isar, Technische Universität München, Bavaria, München 81675 Germany; 5 Department of Stem Cell Biology, School of Medicine, Konkuk University, Seoul, Gwangjin-gu 05029 Republic of Korea; 6 Department of Biomedical Science, CHA University, Seongnam, Bundang-gu 13488 Republic of Korea; 7 Department of Internal Medicine, School of Medicine, Kangwon National University, Chuncheon, Republic of Korea; 8 Department of Pathology, College of Medicine, Hanyang University, Seoul, Seongdong-gu 04763 Republic of Korea

**Keywords:** Differentiation, Lymphangiogenesis, Epigenetics

## Abstract

Proper functioning of the lymphatic system is required for normal immune responses, fluid balance, and lipid reabsorption. Multiple regulatory mechanisms are employed to ensure the correct formation and function of lymphatic vessels; however, the epigenetic modulators and mechanisms involved in this process are poorly understood. Here, we assess the regulatory role of mouse Dot1l, a histone H3 lysine (K) 79 (H3K79) methyltransferase, in lymphatic formation. Genetic ablation of *Dot1l* in Tie2(+) endothelial cells (ECs), but not in Lyve1(+) or Prox1(+) lymphatic endothelial cells (LECs) or Vav1(+) definitive hematopoietic stem cells, leads to catastrophic lymphatic anomalies, including skin edema, blood–lymphatic mixing, and underdeveloped lymphatic valves and vessels in multiple organs. Remarkably, targeted Dot1l loss in Tie2(+) ECs leads to fully penetrant lymphatic aplasia, whereas *Dot1l* overexpression in the same cells results in partially hyperplastic lymphatics in the mesentery. Genetic studies reveal that Dot1l functions in c-Kit(+) hemogenic ECs during mesenteric lymphatic formation. Mechanistically, inactivation of Dot1l causes a reduction of both H3K79me2 levels and the expression of genes important for LEC development and function. Thus, our study establishes that Dot1l-mediated epigenetic priming and transcriptional regulation in LEC progenitors safeguard the proper lymphatic development and functioning of lymphatic vessels.

## Introduction

The lymphatic system plays an important role in immune surveillance and the modulation of fluid balance and lipid reabsorption^1,2^. An important issue in lymphatic biology that remains poorly addressed is the epigenetic mechanisms that coordinate lymphatic endothelial cell (LEC) development and function. Although recent studies suggest a distinct origin for organ-specific LECs^3–6^, the emergence of lineage-committed LECs is generally believed to start with polarized Prox1 expression in a subset of cardinal vein (CV) endothelial cells (ECs) on approximately embryonic day 9.5 (E9.5) in the mouse^7,8^. The development of LECs is tightly controlled via multiple regulatory mechanisms involving transcription factors, such as sex-determining region Y (SRY)-box 18 (Sox18)^9,10^, nuclear receptor subfamily 2, group F, member 2 (Nr2f2, also known as COUP-TFII)^11^, and prospero homeobox 1 (Prox1)^8,12^, as well as the vascular endothelial growth factor C (Vegfc)–vascular endothelial growth factor receptor 3 (Vegfr3) signaling pathway^13–16^. Direct binding of Sox18 to the *Prox1* promoter activates its expression^9^, whereas Nr2f2 physically interacts with Prox1 and modulates its activity^17,18^. The lymphangiogenic factor Vegfr3 has been shown to be necessary for the maintenance of Prox1 expression in LEC progenitors via a positive Prox1–Vegfr3 feedback loop^12^. Lineage-committed LECs bud off from the CV and start migrating toward a high concentration of Vegfc to form primitive lymphatic sacs. A partial or complete blockage of the Vegfc–Vegfr3 axis in LECs causes various lymphatic defects, including aplastic lymphatics in the skin and mesentery, skin edema, and aberrant migration of Prox1(+) LEC progenitors^16,19^. Improper blood–lymph separation due to the malformation of lymphatic valves causes blood–lymphatic mixing. A number of genes involving these processes have been identified, including forkhead box C2 (*Foxc2*)^20,21^, GATA-binding protein 2 (*Gata2*)^22^, *Prox1*^21^, gap junction protein, alpha 4 (*Gja4*)^21,23^, and integrin alpha 9 (*Itga9*)^24^. Studies have demonstrated that genetic mutation or aberrant regulation of the key lymphatic genes are involved in human lymphatic disorders^25,26^.

Furthermore, emerging evidence has suggested that lymphatic development and function may be also subjected to epigenetic regulation^27,28^. Brahma-related gene 1 (Brg1), an ATP-dependent chromatin remodeler, regulates Nr2f2 expression in developing veins. Another chromatin remodeler, chromodomain helicase DNA-binding protein 4 (Chd4), is essential for LEC integrity by regulating urokinase plasminogen activator receptor (*uPAR*) expression^27^. In addition, histone deacetylase 3 (Hdac3) function is required for lymphatic valve formation by regulating *Gata2* expression in response to shear stress^29^. Recently, histone acetyltransferase p300 was shown to promote LEC specification through the activation of lymphatic genes that are critical to the process of blood EC (BEC)-to-LEC differentiation^30^. However, the role of histone methylation in LEC development and function is largely unknown.

Disruptor of telomeric silencing 1-like [Dot1l, also known as lysine methyltransferase 4 (KMT4)] is a histone H3 lysine 79 (H3K79) methyltransferase that plays pivotal roles in the homeostasis of various organs, including the heart^31^ and cartilage^32^, hematopoiesis^33–35^, and cell reprogramming^36^. Previous studies have shown that mistargeting of human DOT1L through its interaction with leukemic fusion proteins is linked to leukemogenesis^37–39^, and that constitutive *Dot1l* knockout (KO) leads to embryonic lethality due to defects in the formation of the extraembryonic vascular network^34,40^. However, little is known about the cell type that causes this vascular phenotype, and whether Dot1l is functionally involved in the formation of other vessel types, including embryonic blood vessels and lymphatic vessels. Here, we demonstrated that epigenetic priming of LEC progenitors by Dot1l confers their precise development and function by controlling the expression of genes important for LEC development and valve formation in the mouse. Therefore, our study established another regulatory mechanism involved in LEC development and function.

## Results

### Dot1l loss in Tie2(+) cells leads to catastrophic lymphatic anomalies

**Fig. 1:**
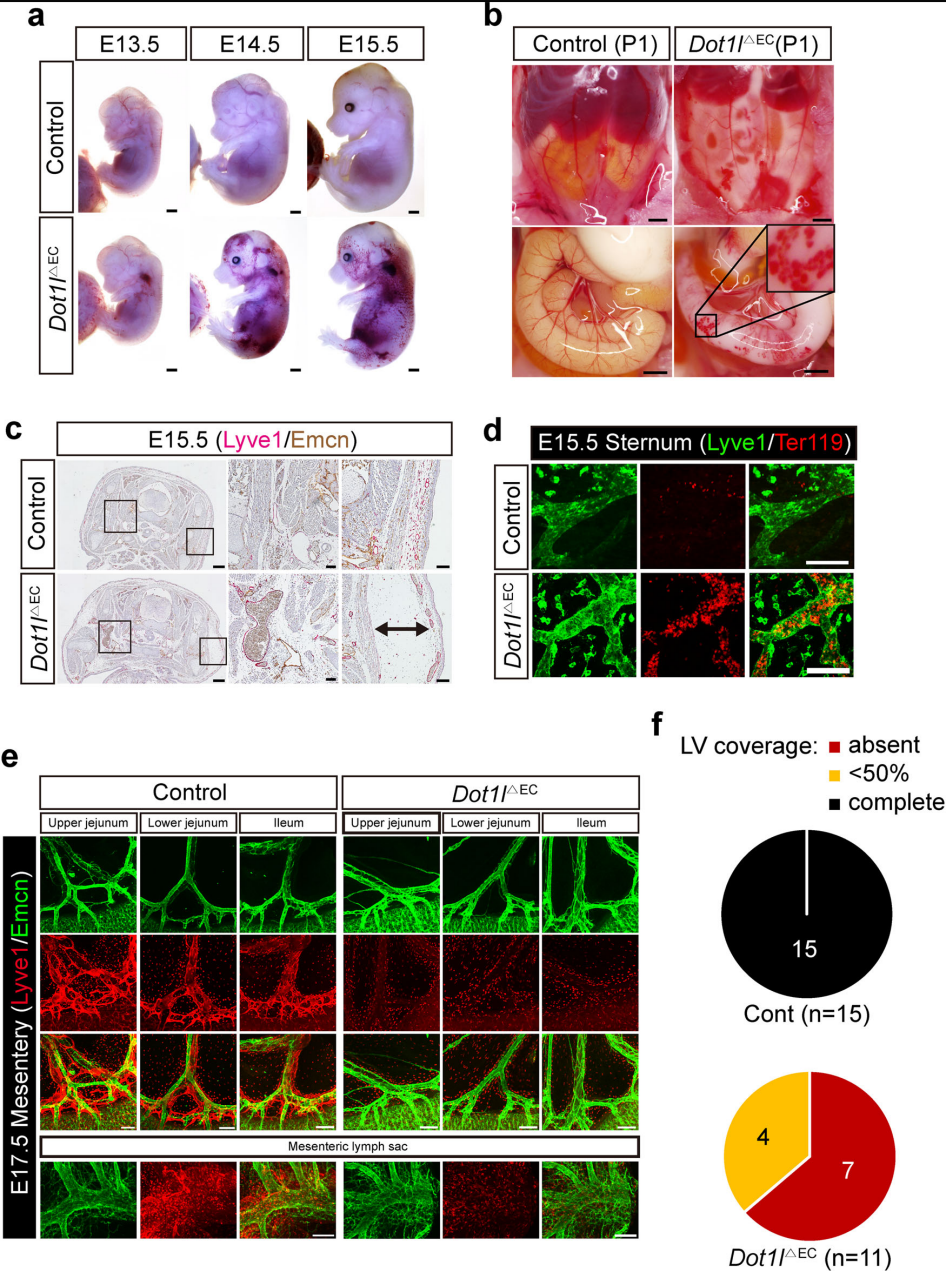
Dot1l depletion in Tie2(+) cells causes lymphatic defects. **a**, **b** Representative images of *Dot1l*^∆EC^ and littermate control embryos at E13.5–15.5 and newborns. Scale bar = 2 mm. P1: postnatal day 1. **c** Immunohistochemistry analysis of E15.5 embryos. Images of Emcn (brown) and Lyve1 (red) in control and *Dot1l*^∆EC^ mice. White rectangles represent enlarged images on right panels. Scale bar = 500 µm. Scale bar of the enlarged image = 100 µm. Arrow: skin edema. **d** Whole-mount confocal images stained with antibodies against Ter119 and Lyve1 in E15.5 sternum. Scale bar = 100 µm. **e** Representative whole-mount confocal images of E17.5 embryos stained with anti-Lyve1 and anti-Emcn antibodies. Lyve1-positive dots: macrophages. Scale bar = 200 µm. **f** Quantification of Lyve1(+) coverage in the mesenteries of E17.5 *Dot1l*^∆EC^ (*n* = 11) and littermate control (*n* = 15) embryos.

Previous studies demonstrated that a Dot1l deficiency caused mid-gestational embryonic lethality, with underdevelopment of yolk-sac vessels and cardiac hypertrophy^31,40^. To gain insight into the function of Dot1l in ECs, embryonic vessel development was assessed in a compound mouse strain carrying *Dot1l*^−/−^;*Tie2*-Cre;*R26R* (Supplementary Fig. S1a, d). Consistent with a previous report, less branched and more disorganized and dilated vessels, as shown by the LacZ reporter, were evident in the mutant brains at E9.5 and 10.5 (Supplementary Fig. S1a, b)^40^. This observation was further confirmed by whole-mount immunostaining of CD31 and quantification of vessel-branching points (Supplementary Fig. S1c, d). To investigate the basis for impaired vessel development, we examined the BEC-autonomous effects of Dot1l function by breeding mice carrying a conditional *Dot1l* allele with a Tg(*Tie2*-Cre) strain, which yielded *Dot1l*^ΔEC^ mice. Unexpectedly, *Dot1l*^ΔEC^ embryos showed normal development by E12.5, without discernible defects in blood vessel formation (Supplementary Fig. S1e–g), suggesting that the blood vessel phenotype observed in E9.5/10.5 *Dot1l*^−/−^ embryos was most likely caused by BEC-independent Dot1l activity. Nevertheless, from E13.5 onward, the *Dot1l*^ΔEC^ animals exhibited lethality, with severe edema and hemorrhage-like spots on the skin, especially on the neck (Fig. 1a). These phenotypes became more severe at later stages, and surviving *Dot1l*^ΔEC^ newborns exhibited chylous ascites (Fig. 1b); none of them survived beyond 3 weeks of age (Supplementary Table S1). Immunohistochemistry and whole-mount immunostaining using anti-Emcn, anti-Ter119, and anti-Lyve1 antibodies revealed that *Dot1l*^ΔEC^ led to skin edema and blood-filled hypoplastic lymphatics in multiple organs, including the heart, diaphragm, and mesentery, at E15.5 and E17.5 (Fig. 1c; Supplementary Fig. 2a–f). Notably, lymphatic aplasia (no or <50% Lyve1(+) lymphatic vessels in 7 and 4 out of 11 embryos, respectively) was observed in the mesentery from the jejunum to the ileum in mutant mice (Fig. 1e, f). The lymphatics in the mesenteric sac of mutant mice were also hypoplastic compared with those in control mice (Fig. 1e). The lymphatic phenotype in the mesentery was reconfirmed by whole-mount immunofluorescent stainings with other LEC markers including PROX-1, NRP2 and VEGFR3 (data not shown). However, a loss of Dot1l had little effects on skin lymphatic vessel formation (Supplementary Fig. S2g, h). Together, these data suggest that a loss of Dot1l in cells that historically express Tie2 causes defects in lymphatic vessels rather than blood vessels.

### *Dot1l*^ΔEC^ impairs the formation of lymphatic valves

**Fig. 2:**
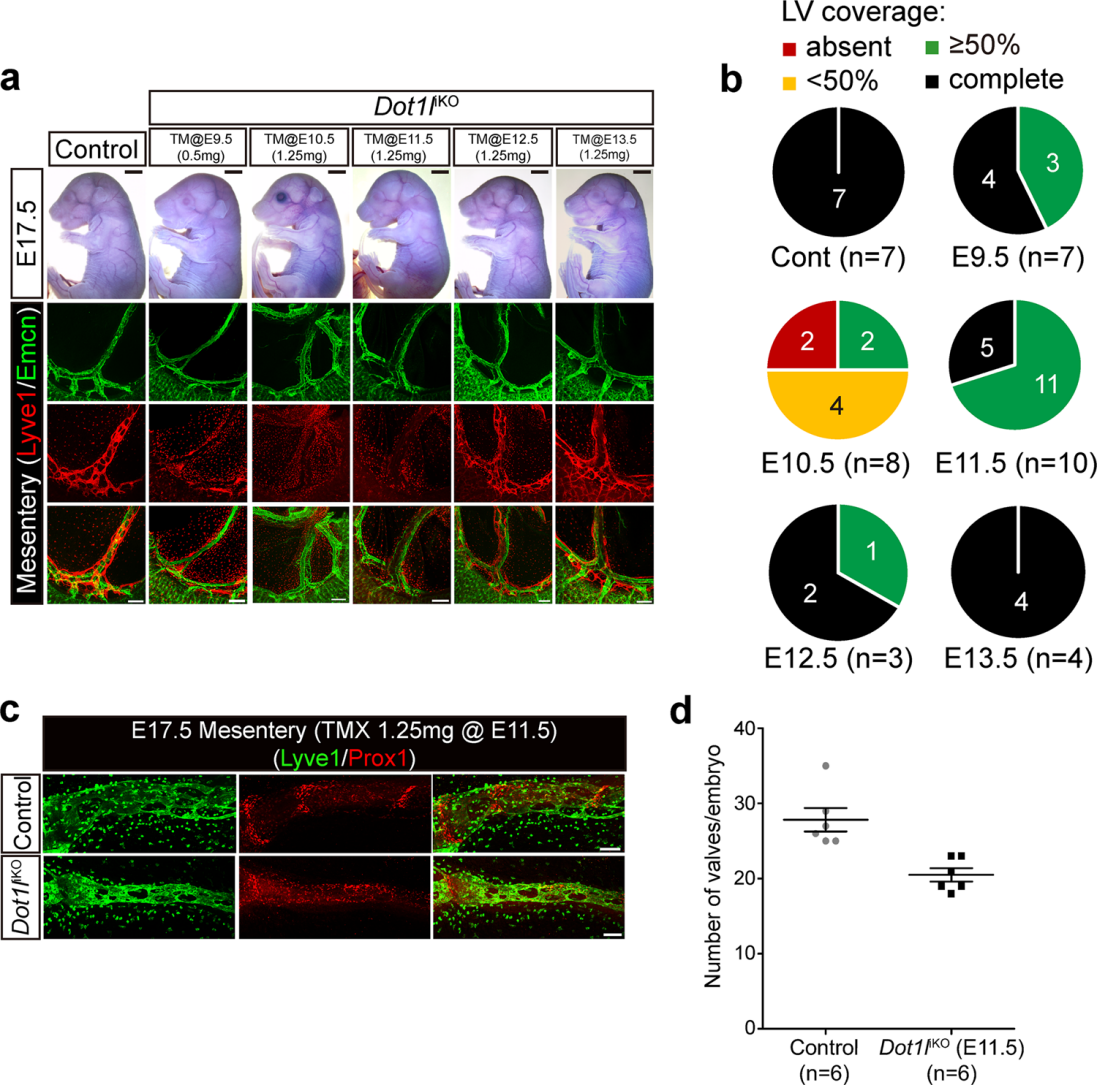
Dot1l loss impairs lymphatic valve formation. **a** Gross overview of E17.5 embryos (upper panel, scale bar = 2 mm) and their whole-mount immunofluorescent staining with anti-Lyve1 and anti-Emcn antibodies in the respective mesenteries (lower panel) after a single TM injection (0.5 mg for E9.5 and 1.25 mg for E10.5 embryos). Scale bar = 200 µm. **b** Quantification of Lyve1(+) lymphatic coverage in TM-injected E17.5 *Dot1l*^iKO^ mesenteries (*n* = 3–16 embryos/group). **c**, **d** Representative immunofluorescence images and morphometric analysis of lymphatic valves in E17.5 *Dot1l*^iKO^ mesenteries after TM injection at E11.5. Green: Lyve1. Red: Prox1. Data are presented as mean ± s.e.m. *p-value ≤ 0.05.

Given that aberrant lymphatic valve formation can cause blood–lymphatic mixing, we next sought to determine the function of Dot1l in lymphatic valve formation in a strain other than the *Dot1l*^ΔEC^ mice as the formation of lymphatics in multiple organs is impaired in the mice. Therefore, *Dot1l* was temporally abolished by using a robust inducible Cre driver, *ROSA26*-CreER, in *Dot1l*^iKO^ mice, and the mesenteric lymphatics were examined. Since constitutive KO of *Dot1l* affects embryo viability, we first determined the doses of tamoxifen (TM) that had minimal effects on embryonic survival; the optimal doses were 0.5 mg/25 g for E9.5 embryos and 1.25 mg/25 g for E10.5–13.5, since injection of the higher dose (1.25 mg/25 g) on E9.5 caused complete embryonic lethality by E14.5–15.5. Nearly half of the E17.5 mutant embryos displayed hypoplastic mesenteric lymphatics after a single injection of the low dose (0.5 mg/25 g) at E9.5 (in three out of seven embryos with ≥50% coverage), whereas at the higher TM dose, severe and frequent lymphatic hypoplasia was detected in the mesentery at E10.5 (in six out of eight embryos with <50% coverage and in two out of eight embryos with ≥50% coverage). The phenotype was alleviated when this dose of TM was injected at later stages (in seven out of ten embryos at E11.5, one out of three embryos at E12.5, and none at E13.5) (Fig. 2a, b). Then, to facilitate the assessment of Tie2(+) cells, in which Dot1l regulates lymphatic valve formation, E17.5 mesenteries were harvested from the E11.5 TM-injected *Dot1l*^iKO^ mice and analyzed by immunofluorescence with anti-Prox1 and anti-Lyve1 antibodies followed by morphometric analysis. As shown in Fig. 2c, d, a significantly reduced number of lymphatic valves was detected in the *Dot1l*^iKO^ mesenteric lymphatics.

### Dot1l priming in LEC progenitors is required for proper LEC development

**Fig. 3:**
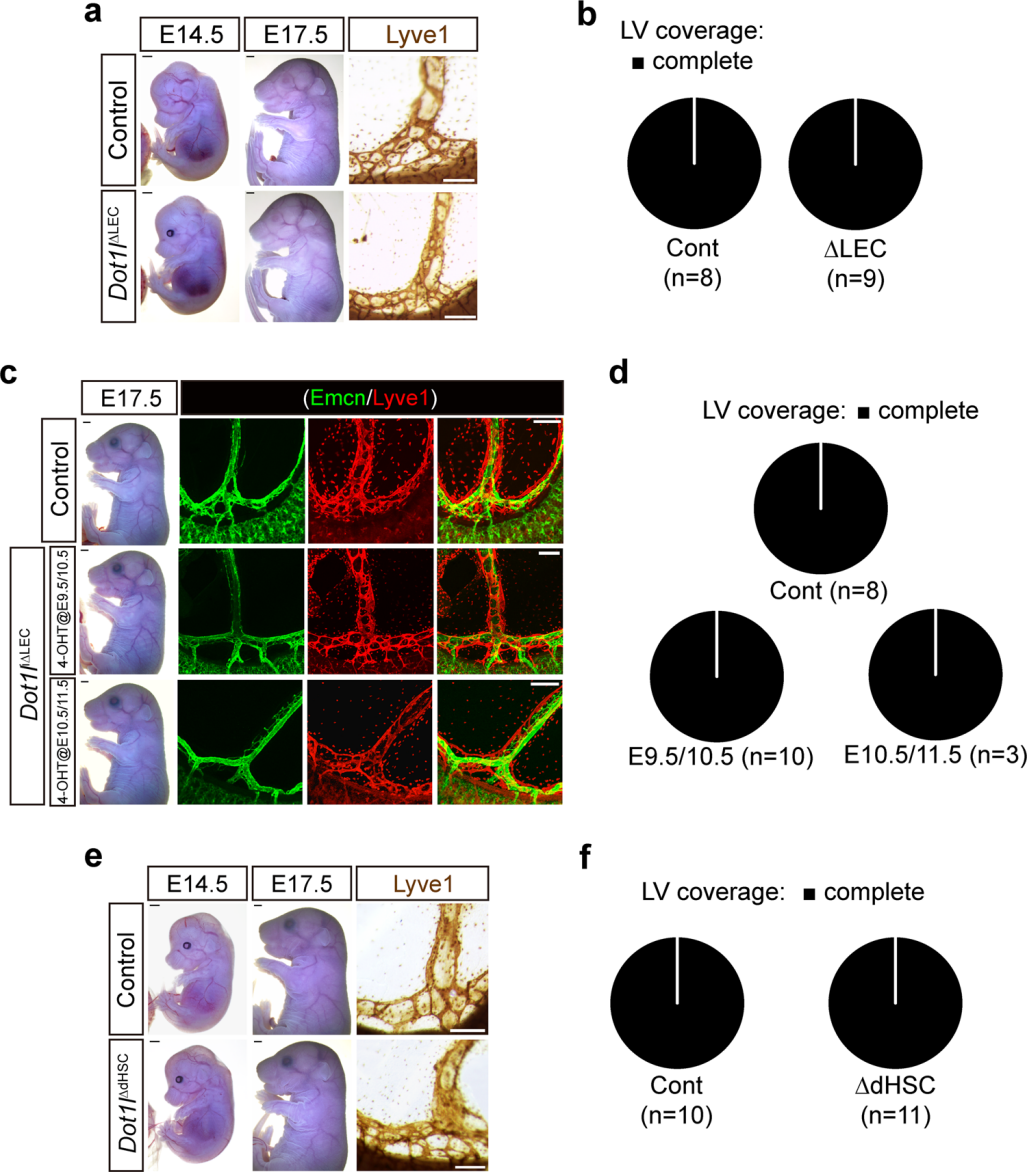
Dot1l loss in Lyve1(+), Prox1(+), or Vav1(+) cells does not cause LEC defects. **a**, **c**, **e** Representative images of embryo (scale bar = 2 mm) and Lyve1-stained mesenteric lymphatics (scale bar = 200 µm): *Dot1l*^∆LEC^ (**a**, E14.5 and 17.5), *Dot1l*^i∆LEC^ (**c**, E17.5), and *Dot1l*^∆dHSC^ (**e**, E14.5 and 17.5). **b**, **d**, **f** Quantification of Lyve1(+) lymphatic vessel coverage in E17.5 *Dot1l*^∆LEC^ (**b**, control = 8, KO = 9) and *Dot1l*^i∆LEC^ (**d**, control = 8, KO = 10 and 3) embryos after treatment with 4-OHT at E9.5/10.5 and E10.5/11.5, and in E17.5 *Dot1l*^∆dHSC^ (**f**, control = 10, KO = 11) embryos.

Since Tie2(+) cells can develop into LECs, HSCs, and BECs, we next determined whether the lymphatic abnormality observed in the *Dot1l*^ΔEC^ mice was caused by a Dot1l deficiency in LECs or HSCs. To this end, mice carrying a conditional *Dot1l* allele were crossed with an LEC-specific Cre driver, *Lyve1*^EGFP/Cre^, to generate a *Dot1l*^ΔLEC^ strain (Fig. 3a). Interestingly, *Lyve1*^EGFP/Cre^-mediated Dot1l depletion caused neither embryonic lethality nor the lymphatic phenotypes observed in the *Dot1l*^ΔEC^ mice (Fig. 3b). *Dot1l*^ΔLEC^ mice were born at the expected Mendelian ratio and appeared healthy during the postnatal period. The absence of a lymphatic phenotype in *Dot1l*^ΔLEC^ is not due to an inefficient Cre recombinase, as *Lyve1*^EGFP/Cre^ displays the expected Cre activity in a subset of E10.5 CV BECs and adjacent LECs, and E17.5 mesenteric lymphatics (Supplementary Fig. S3a, b). To confirm this observation, mice carrying the *Dot1l* conditional allele were bred with another LEC-specific inducible Cre driver, Tg(*Prox1*-CreER^T2^), to generate the *Dot1l*^iΔLEC^ strain. None of E17.5 *Dot1l*^iΔLEC^ embryos displayed the lymphatic defects observed in the *Dot1l*^ΔEC^ mice after 4-hydroxytamoxifen (4-OHT) administration for two consecutive days on E9.5/E10.5 or E10.5/E11.5 (Fig. 3c, d).

Given that Vav1(+) dHSCs contribute to the development of cardiac lymphatics^3^, we next examined the effect of Dot1l depletion in dHSCs on LEC development using a Tg(*Vav1*-iCre) strain (*Dot1l*^ΔdHSC^). None of the mutant embryos exhibited defects in the skin or mesenteric lymphatics (Fig. 3e, f), which is consistent with a previous report showing that an independent *Dot1l*^ΔdHSC^ strain was viable but showed impaired hematopoiesis^37^. These results support the notion that a loss of function in BECs/LEC progenitors, but not in the LECs or dHSCs, mediates the observed lymphatic defects.

### Dot1l function in c-Kit(+) hemogenic ECs is required for mesenteric LEC development

**Fig. 4:**
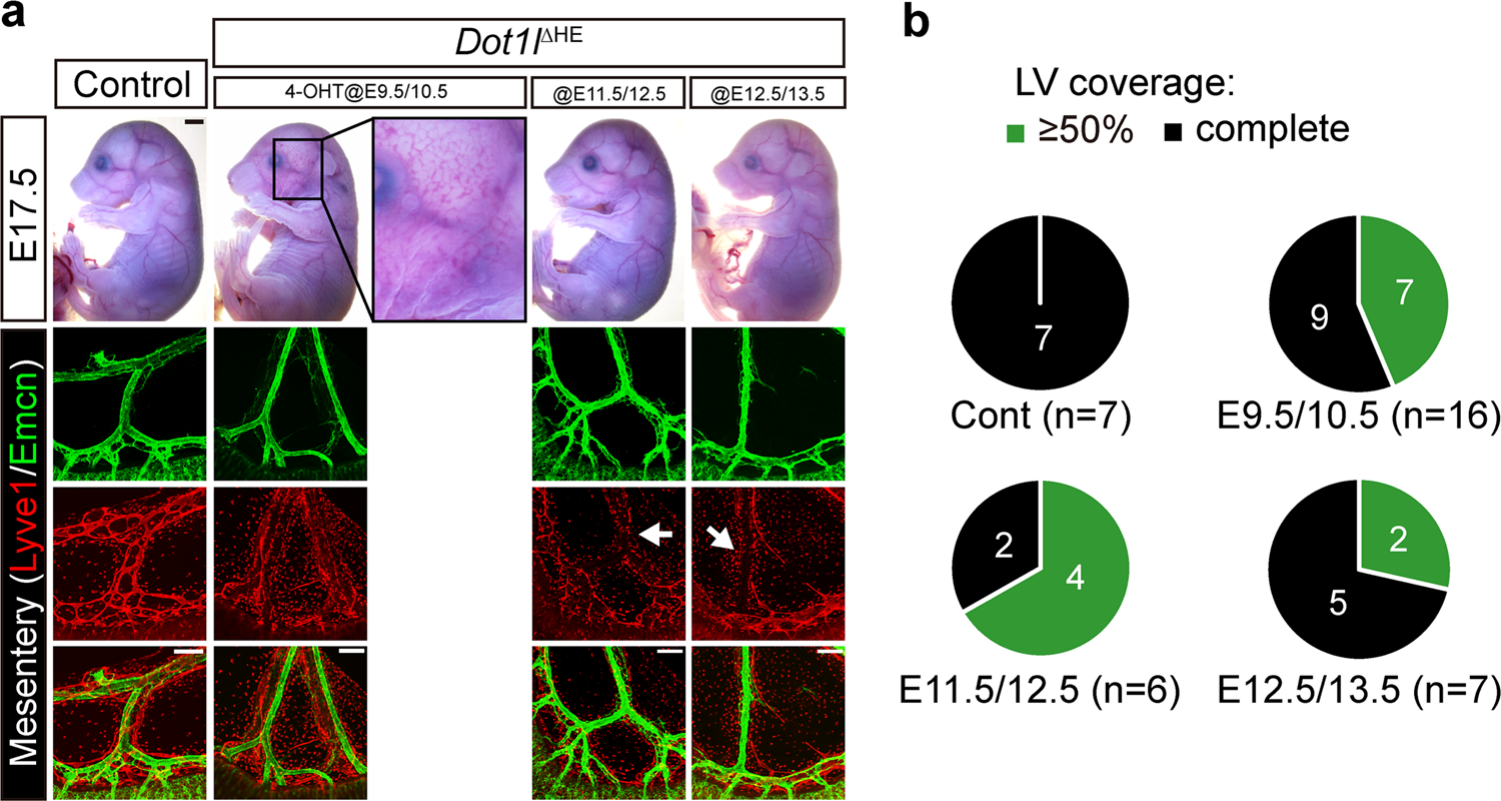
Dot1l function in c-Kit(+) HE is indispensable for the formation of mesenteric lymphatics. **a** Representative images of E17.5 embryos (upper panel, scale bar = 2 mm) and whole-mount immunofluorescence staining of *Dot1l*^∆HE^ mesenteries with anti-Lyve1 and anti-Emcn antibodies (lower panel, scale bar = 200 µm) after two consecutive 4-OHT injections. Incomplete lymphatic formation (arrows) is indicated. **b** Quantification of Lyve1(+) coverage in E17.5 *Dot1l*^∆HE^ and control lymphatic vessels (*n* = 6–16 embryos per group).

A recent study revealed that the formation of mesenteric lymphatics is mediated by both lymphangiogenesis of preexisting lymphatics from CV BECs and lymphvasculogenesis of c-Kit(+) HEs, which presumably originate from both the yolk sac and aorta–gonad–mesonephros (AGM)^4^. Evidence also suggests that cardiac LECs partly originate from yolksac-derived HEs^3^. Moreover, the most severe lymphatic phenotype was observed when Dot1l was abolished at the time when HEs are actively formed in the mesentery of *Dot1l*^iKO^ mice (Fig. 2a, b). Thus, to directly evaluate the requirement of Dot1l in c-Kit(+) HEs for the regulation of mesenteric LEC differentiation, we examined the mesentery in E17.5 *Dot1l*^2fl/2fl^;*cKit*^CreERT2^ (*Dot1l*^ΔHE^) embryos after 4-OHT injection for two consecutive days on E9.5/E10.5, E11.5/E12.5, or E12.5/E13.5. Incomplete formation of the mesenteric lymphatics was observed in a subset of E17.5 *Dot1l*^ΔHE^ embryos (7 out of 16 injected on E9.5/E10.5 and 4 out of 6 injected on E11.5/E12.5), whereas milder lymphatic defects were detected following injection on E12.5/E13.5 (2 out of 7 embryos) (Fig. 4a, b). Taken together, these results strongly suggest that epigenetic priming by Dot1l in LEC progenitors, including both Tie2(+) and c-Kit(+) cells, during LEC differentiation is essential for the formation of mesenteric lymphatics. Moreover, given that HEs from the yolk sac are Lyve1 positive, whereas HEs from AGM are Lyve1 negative^41^, our findings also imply that Dot1l function in yolk-sac-derived HEs may have little effect on mesenteric LEC differentiation.

### Dot1l depletion alters the lymphatic transcription program

**Fig. 5:**
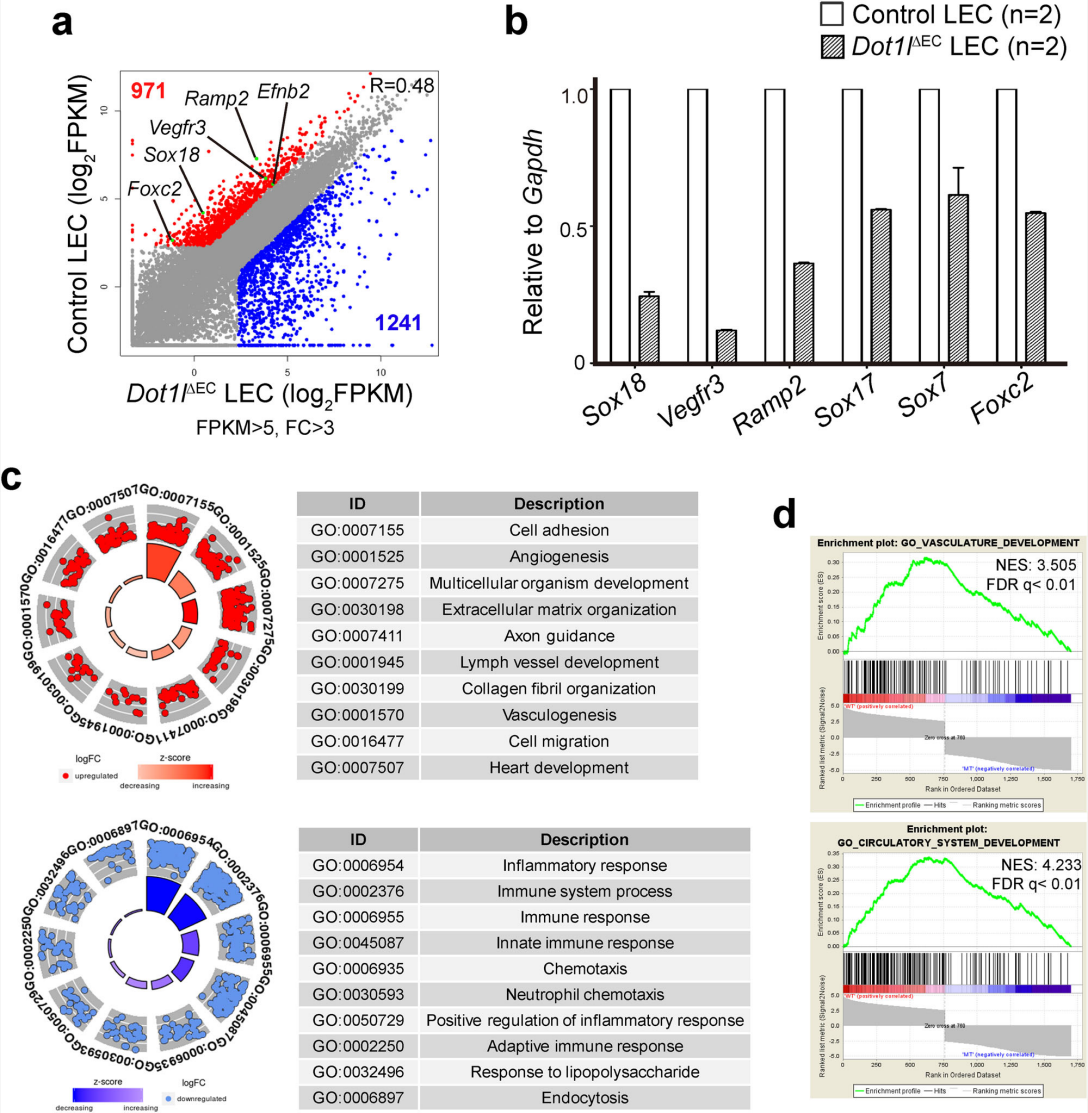
Dot1l loss impairs transcriptional program important for LEC development and function. **a** Scatter plot of RNA-Seq analysis of LECs isolated from E15.5 control (*n* = 3) and *Dot1l*^∆EC^ (*n* = 2) embryos. *n* = 2 and 3: pooled biological replicates per RNA-Seq library. Genes critical for lymph vessel formation and function are indicated. Red and blue dots indicate downregulated and upregulated genes in *Dot1l*^∆EC^ LECs, respectively. **b** qRT-PCR analysis confirming reduced gene expression in E15.5 *Dot1l*^∆EC^ dermal LECs compared with the wild type (*n* = 2/group). Error bars show mean ± s.e.m. **c**, **d** Gene Ontology (GO) term analysis and Gene Set Enrichment Analysis (GSEA) with genes downregulated in *Dot1l*^∆EC^ LECs. Each red (repressed genes in cKO) and blue (upregulated genes in cKO) represents one gene in each GO term, respectively. NES normalized enrichment score, FDR false discovery rate.

To understand the mechanism underlying Dot1l-mediated regulation of LEC development, RNA sequencing (RNA-Seq) of LECs isolated from E15.5 *Dot1l*^ΔEC^ skin was performed. The results indicated that 971 and 1241 genes were downregulated and upregulated in *Dot1l*^ΔEC^ LECs, respectively (Fig. 5a). Importantly, many genes known to be critical for lymphatic development and valve formation were downregulated in KO LECs, including *Sox18*^9,10^, *Vegfr3*^12,14,15^, *Ramp2*^42^, and *Foxc2*^20,21^ (Fig. 5a), which was further confirmed by qRT-PCR (Fig. 5b). Gene Ontology (GO) and Gene Set Enrichment Analysis (GSEA) analyses of the genes repressed by Dot1l conditional knockout (cKO) revealed marked enrichment of genes involved in both blood and lymphatic vessel development (Fig. 5c, d). Interestingly, groups of genes related to immunity were significantly upregulated in *Dot1l*^ΔEC^ LECs (Fig. 5c).

### Dot1l inactivation reduces H3K79me2 enrichment at lymphatic genes

**Fig. 6:**
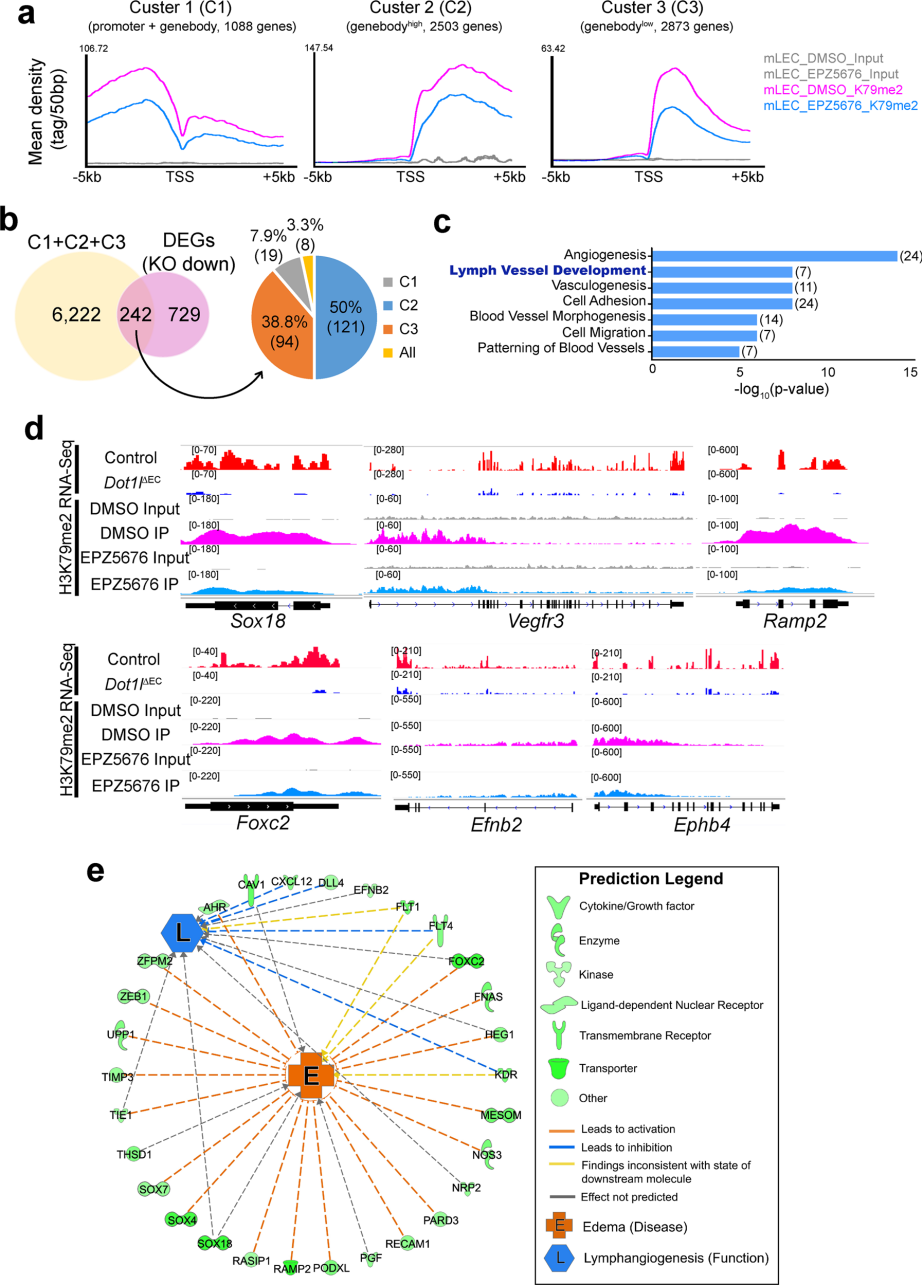
Dot1l directly regulates expression of key genes associated with LEC development and function. **a** Mean tag density plots showing k-means clustering of H3K79me2 enrichment in DMSO- or EPZ5676-treated mouse primary dermal LECs (*n* = 3, 3: pooled biological replicates per ChIP-Seq library). Based on the pattern of H3K79me2 enrichments, peaks are subcategorized into cluster 1 (promoter + genebody, 1088 genes), cluster 2 (genebody^high^, 2503 genes), and cluster 3 (genebody^low^, 2873 genes). H3K79me2 peaks in the region from –5 to +5 kb around the transcription start site (TSS) are shown. **b** Venn diagram and pie chart showing the number of genes commonly downregulated in both expression and H3K79me2 levels in each gene cluster. **c** GO term analysis of the common 242 genes. The number of genes in each term is indicated in parenthesis. –log_10_(*p*-value) was used for the bargraph. **d** Genome browser view of downregulated Dot1l target genes (*Sox18*, *Vegfr3, Ramp2, Foxc2, Efnb2*, and *Eph4*) crucial for LEC development. **e** Ingenuity Pathway Analysis (IPA) of the genes identified as reduced by both expression and H3K79me2 ChIP-Seq analyses. Note that the genes associated with aberrant lymphangiogenesis and edema in mouse and human were significantly downregulated by Dot1l loss. Each shape and line color in the legend represents protein function and functional interaction, respectively.

To determine the direct target genes of Dot1l, H3K79me2 ChIP-Seq was performed in LECs exposed to the DOT1L inhibitor EPZ5676. Our analysis revealed that inactivation of Dot1l caused a significant reduction in H3K79me2 levels at cluster 1 (promoter+genebody, 1088 genes), cluster 2 (genebody^high^, 2503 genes), and cluster 3 (genebody^low^, 2873 genes) (Fig. 6a). Importantly, 242 genes (19 in cluster 1, 121 in cluster 2, 94 in cluster 3, and 8 in cluster 1 + 2 + 3) involved in angiogenesis, lymph vessel development, and vasculogenesis showed downregulated H3K79me2 levels and gene expression (Fig. 6b, c). Notably, a reduction in genebody H3K79me2 was observed for most of the commonly repressed genes (Fig. 6b). Representative genes that are important for LEC differentiation, migration, and valve formation were visualized in the IGV genome browser (Fig. 6d). These genes include *Sox18*, *Vegfr3*, *Ramp2*, *Foxc2*, *Efnb2*, and *Ephb4*. Ingenuity pathway analysis (IPA) revealed that a subset of the genes commonly repressed by Dot1l inactivation were associated with edema and aberrant lymphangiogenesis (Fig. 6e). Taken together, Dot1l-mediated H3K79 methylation in LECs contributes to the proper expression of genes that are important for lymphatic vessel formation and function during LEC development.

### Dot1l overexpression in Tie2(+) or Lyve1(+) cells leads to aberrant lymphatic formation

**Fig. 7:**
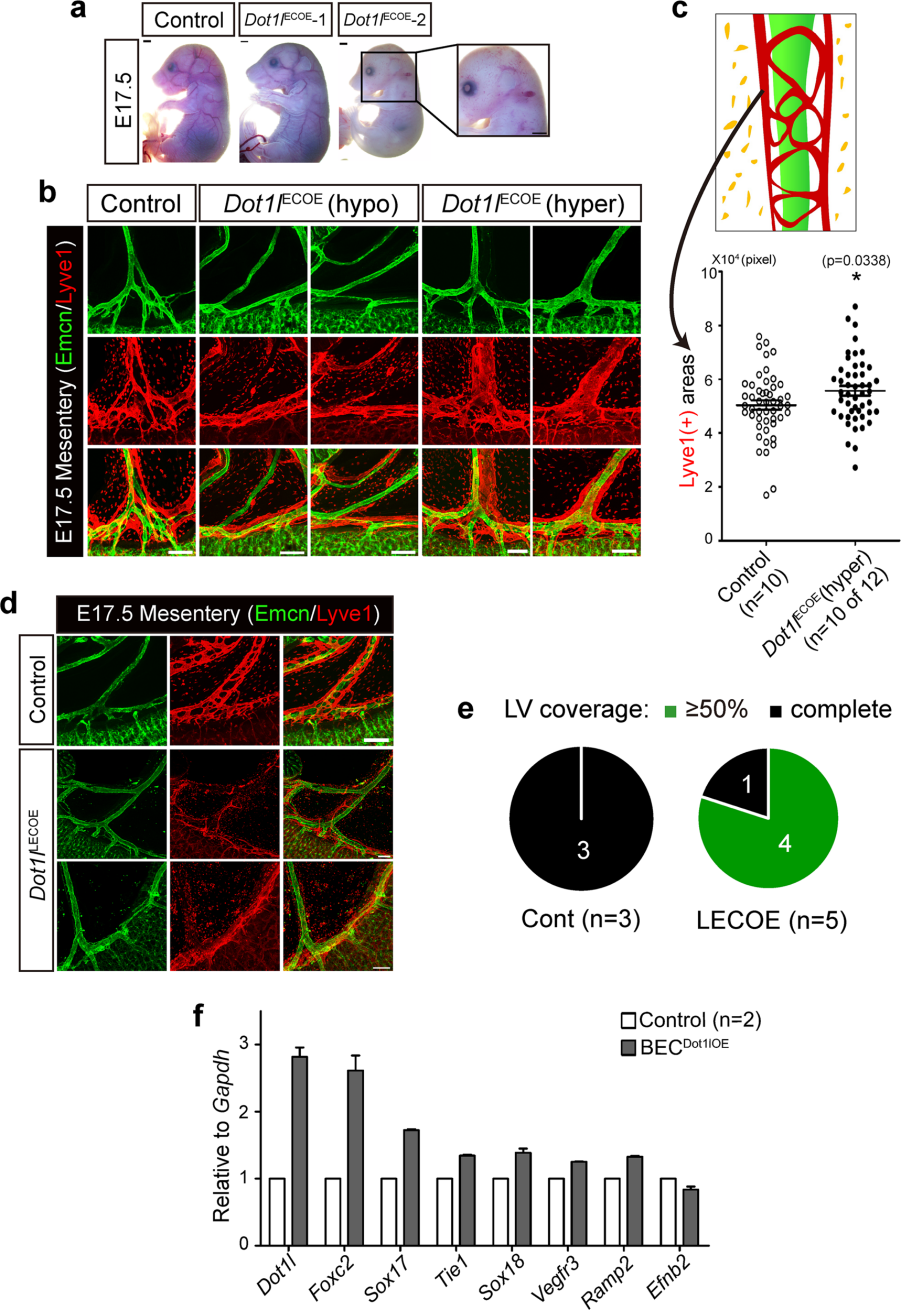
Targeted *Dot1l* overexpression in Tie2(+) or Lyve1(+) cells causes aberrant lymphatic formation. **a** Gross overview of E17.5 embryos (scale bar = 2 mm). Enlarged image shows lymphatic–blood mixing in *Dot1l*^ECOE^ skin. **b** Whole-mount immunofluorescence with anti-Lyve1 and anti-Emcn antibodies in *Dot1l*^ECOE^ mesenteries. Scale bar = 200 µm. **c** Morphometric analysis of mesenteric lymphatics in E17.5 *Dot1l*^ECOE^ (*n* = 10) and littermate control embryos (*n* = 10). Error bars show mean ± s.e.m. **d** Representative immunofluorescence with anti-Lyve1 and anti-Emcn antibodies in *Dot1l*^LECOE^ mesenteries. **e** Morphometric analysis of Lyve1(+) coverage of mesenteric lymphatics in E17.5 *Dot1l*^LECOE^ (*n* = 5) and littermate control embryos (*n* = 3). **f** qRT-PCR analysis of lymphatic genes in BECs overexpressing *Dot1l*. *n* = 2.

To complement the loss-of-function studies, we created a novel knock-in (KI) mouse strain in which mouse *Dot1l* cDNA*-IRES-EGFP* with a floxed 3 × poly(A) was inserted into the *ROSA26* locus (Supplementary Fig. S4a, b). These KI mice were crossed with the Tg(*Tie2*-Cre) line to obtain a compound strain overexpressing *Dot1l* in ECs (*Dot1l*^ECOE^). Cre-mediated excision of the poly(A) signal followed by transgene expression in ECs was validated by evaluating *EGFP* expression in E17.5 *Dot1l*^ECOE^ mesenteric vessels (Supplementary Fig. S4c). Mesenteric lymphatic vessel enlargement, especially in the ileal mesentery, was evident in 10 out of 12 E17.5 m*Dot1l*^ECOE^ embryos (Fig. 7a–c). Intriguingly, 2 out of 12 embryos and 1 out of 12 embryos displayed hypoplastic mesenteric lymphatics and the blood–lymphatic mixing phenotype in the skin, respectively (Fig. 7a, b). Next, to address which EC type was responsible for the phenotype observed in m*Dot1l*^ECOE^ mice, an m*Dot1l*^LECOE^ strain was generated using the *Lyve1*^EGFP/Cre^ line. Unlike in m*Dot1l*^ECOE^ mice, discontinuous and hypoplastic lymphatics were observed in the mesentery of E17.5 m*Dot1l*^LECOE^ mice (Fig. 7d, e). These data indicate that Dot1l plays a distinct role depending on cell type (i.e., before or after LEC differentiation). Finally, we sought to determine whether increased *Dot1l* expression in BECs could enhance the repression of lymphatic genes upon Dot1l loss. To that end, we took advantage of catalytically dead Cas9 (dCas9) to activate endogenous *Dot1l* expression in mouse primary skin BECs (Supplementary Fig. S4d). As shown in Fig. 7f, forced *Dot1l* overexpression led to moderate enhancement of key lymphatic genes, such as *Foxc2*, *Sox17*, *Tie1*, *Sox18*, *Vegfr3*, and *Ramp2* on day 7 post transduction. Collectively, our analysis revealed that meticulously regulated Dot1l function in BECs or lymphatic progenitors is critical for normal LEC differentiation and lymphatic development.

## Discussion

The formation and maintenance of functional lymphatic circulation are key for mammalian physiology. In this study, epigenetic priming by Dot1l in LEC progenitors was found to play an essential role in lymphatic vessel development and valve formation. Dot1l is the only known H3K79 methyltransferase that does not contain a canonical histone methyltransferase domain, referred to as the Su(var)3-9, Enhancer of Zeste and Trithorax (SET) domain^19,43,44^. Studies showed that Dot1l is enriched at actively transcribed genes through its interaction with phosphorylated C-terminal domain of RNA polymerase II (PolII). Thus, enrichment of di- and trimethylated H3K79 (H3K79me2/3) in genebodies is positively correlated with PolII elongation and transcription efficiency^45–47^. Consistently, our genome-wide analysis indicated that Dot1l directly binds to critical lymphatic genes that coordinate lymphatic development and function. The most prominent phenotypes observed in Dot1l cKO animals are lymphatic hypoplasia, edema, and underdevelopment of lymphatic valves. The phenotypes described in this study are consistent with previous KO studies. It was previously shown that KO of Sox18 and Foxc2 perturbs LEC differentiation from BECs, leading to aplastic lymphatics and lymphatic valve formation, respectively^9,20,21^. Accumulating evidence suggests that DOT1L has context-dependent beneficial or adverse effects on human disease. For example, DOT1L promotes the progression of neuroblastoma, whereas it protects against the development of UV-induced melanoma^48,49^. Nguyen et al. demonstrated that Dot1l function is essential for the normal maintenance of cardiovascular homeostasis, as a loss of Dot1l function in cardiomyocytes led to dilated cardiomyopathy, with repressed dystrophin expression^31^.

The genes showing repressed H3K79me2 occupancy and expression following Dot1l inactivation included several transcription factors (Sox18, Sox17, and Foxc2) that are critical for LEC differentiation and valve formation, and a signaling molecule (Vegfr3) that is critical for LEC proliferation and migration^9,10,16,20,21^. Mutation of human SOX18 is associated with hypotrichosis–lymphedema–telangiectasia (OMIM 607823), which is characterized by lower-limb lymphedema, cutaneous telangiectasia, and dilatation of superficial vessels^50,51^. In addition, mutations in VEGFR3 and FOXC2 are linked to rare lymphatic disorders called Nonne–Milroy lymphedema (OMIM 153100) and lymphedema–distichiasis syndrome (LDS, OMIM 153400), respectively^52–55^. Patients with Nonne–Milroy lymphedema or LDS also display severe lymphedema, especially in the lower limbs. Consistent with the pathological characteristics of these disorders, *Dot1l*^ECKO^ mice exhibit severe skin edema with impaired lymphatic valve formation. It is generally thought that the MAPK signal initiates venous EC-to-LEC transdifferentiation via transcriptional activation of Sox18, which subsequently induces Prox1 expression^56,57^. Then, Prox1 can form a heterodimeric complex with Nr2f2 to modulate the expression of multiple lymphatic genes, including Vegfr3 and Pdpn^17,18^. Vegfc/Vegfr3 signaling was shown to be indistinguishable for centrifugal lymphangiogenesis by promoting LEC proliferation, migration, and survival^16^. However, the epigenetic regulation of the core transcription factors involved in the development and functioning of lymphatics is poorly understood. To the best of our knowledge, ours is the first study to show that histone methylation is a critical contributor to LEC development through direct regulation of transcription factors and signal transduction. Similarly, a recent study demonstrated that histone acetylation plays critical roles in LEC development and function. Regulation of histone acetylation by elevated fatty acid β-oxidation (FAO) in lineage-committed LEC progenitors ensures proper gene expression for LEC differentiation and function^30^. FAO enhancement is mediated by Prox1-targeted Cpt1a expression and leads to the production of mitochondrial acetyl-CoA, which can function as a cofactor of p300-mediated histone acetylation. Loss of function of mouse Hdac3 reduced lymphatic valve formation and blood–lymphatic mixing, with aberrant gene transcription^29^. Therefore, our data and others suggest that transcriptional control by epigenetic mechanisms is essential for lymph vessel formation and function.

Recent evidence has suggested the possibility that organ-type-specific LECs may not be of a single origin, and instead may be diverse in origin^3–6^. In particular, at least a portion of the heart and mesenteric LECs are derived from the yolk sac and/or the AGM HEs^3,4^. Our finding further supports that Tie2(+)/c-Kit(+)/Vav1(–) HEs likely give rise to mesenteric LECs, as a lymphatic defect was evident in both *Dot1l*^ECKO^ and *Dot1l*^ΔHE^ mice. It is interesting to note that the hypoplastic lymphatic phenotype in the *Dot1l*^ECKO^ embryos is not due to apoptosis in LECs, as we failed to detect an increase in cleaved caspase-3-positive LECs (data not shown). However, it is unclear why and how a loss of Dot1l has little or no effect on BEC development and function in *Dot1l*^ECKO^ mice. There are several possible explanations. First, although Dot1l is broadly expressed, our RNA-Seq analysis with public data showed that Dot1l is more abundantly expressed in AGM c-Kit(+)/CD31(+) cells than in c-Kit(–)/CD31(+) cells, suggesting a critical role for Dot1l in HEs (data not shown). Second, although no other H3K79 demethylase has been identified, it is also feasible that H3K79 methylation and demethylation are much more dynamic in BECs than in LECs, due to the activity of a yet unknown H3K79 demethylase. Indeed, H3K79 methylation seems to be actively reversible. Alternatively, an unknown H3K79 demethylase may be highly expressed in BECs compared with LECs. Lastly, previous studies showed that Dot1l can interact with various binding partners to form a protein complex. These proteins include MLL fusion partners (AF4, AF9, AF10, and ENL) and P-TEFb, which is a kinase of RNA PolII^58,59^. AF17 was shown to modulate Dot1l-mediated placement of H3K79me2 and interfere with Dot1l trafficking into the nucleus by competing for AF9 binding, respectively. Therefore, it is also possible that unidentified Dot1l-interacting proteins are differentially expressed in either cell type to modulate Dot1l activity or the accessibility of target chromatins.

Our *Dot1l* overexpression study revealed that Dot1l-mediated epigenetic regulation has distinct cell-type- and time-dependent effects; *Dot1l* overexpression before/during LEC differentiation exhibited hyperplastic lymphatics in the mesentery, whereas *Dot1l* overexpression after LEC differentiation exhibited hypoplastic lymphatics.

In summary, our demonstration that Dot1l controls transcriptional circuits in the lymphatic system, provides a basis for developing better therapeutic strategies to treat DOT1L-related leukemic patients, especially those with pregnancy. Furthermore, our results suggest *DOT1L* as a candidate biomarker for genetic screening to identify the cause of idiopathic lymphatic disorders including chylous ascites and lymphedema.

## Materials and methods

### Mice

All animal studies were reviewed and approved by Institute of Animal Care and Use Committee (IACUC) of Gachon University (IACUC#LCDI-2014-0045), CHA University (IACUC#180001), and Konkuk University (IACUC#KU18027). Generation of *Dot1l* KO and conditional allele mice was described in a previous study^40^. Tg(*Tie2*-Cre) (stock # 004128), Tg(*Vav1*-iCre) (stock # 008610), *Lyve1*^EGFP/Cre^ (stock # 012601), *ROSA26*^CreER^ (stock # 004847), and R26R (stock # 003474) mice were purchased from Jackson Laboratory (Bar Harbor, USA). Generation of Tg(*Prox1*-CreER^T2^)^60^ and *cKit*^CreERT2^ mice^61^ was described in previous studies. To obtain *Dot1l*^*Δ*EC^, *Dot1l*^ΔLEC^, *Dot1l*^iΔLEC^, *Dot1l*^ΔdHSC^, *Dot1l*^iKO^, and *Dot1l*^ΔHE^ embryos, female *Dot1l*^2f/l2fl^ mice were crossed with male *Dot1l*^2fl/+^;Cre(+) or *Dot1l*^2f/l2fl^;CreER(+) mice; littermate *Dot1l*^2fl/2fl^;Cre(–) or CreER(–) embryos were used as control and *Dot1l*^2fl/2fl^;Cre(+) or CreER(+) embryos were used as the experimental group. For timed mating, vaginal plug was examined at noon and embryos were harvested at designated embryonic days.

To generate the *Dot1l* overexpression allele, 4.6 kb of full-length mouse *Dot1l* cDNA was cloned into pBSApBpACAGftIGn vector^62^ at the *Sfi*I sites, and *Pac*I–*Asc*I fragment from the pBSApBpACAGftIGn vector was subcloned into pROSA26PAS^63^ vector containing *CAGG* promoter and *IRES*-*EGFP*. After electroporation and puromycin selection, genomic DNA was extracted from embryonic stem cells, digested with *Eco*RI, and analyzed by Southern blotting; the expected sizes of *Eco*RI digestion fragments for the knock-in (KI) and wild-type alleles were 6.8 and 15.6 kb, respectively. A standard protocol was used for generation of *Dot1l* KI chimeric mice^64^. To obtain m*Dot1l*^LECOE^ and m*Dot1l*^ECOE^ strain, the KI female mice were crossed with male *Lyve1*^EGFP/Cre^ and Tg(*Tie2*-Cre) lines, respectively.

For genotyping, yolk sacs of embryos or tail tips from embryos/animals were lysed in 25 mM NaOH for 2 h at 95 °C. After neutralization with 1 M Tris-Cl, the lysates were centrifuged at the maximum speed, and supernatants containing genomic DNA were used as templates for PCR. Amplification was carried out under the following conditions: denaturation for 5 min at 95 °C followed by 35 cycles of denaturation for 30 s at 95 °C, annealing for 30 s at 58 °C, and extension for 30 s at 72 °C. Sequences of PCR primers used for genotyping are shown in Table EV2.

To induce Cre activity, tamoxifen (T5648, Sigma) dissolved in corn oil (0.5 mg/25 g or 1.25 mg/25 g) or 4-hydroxytamoxifen (H6278, Sigma) dissolved in DMSO (2 mg/25 g) were injected intraperitoneally into pregnant females, and embryonic organs were harvested at the designated days.

### Whole-mount staining, imaging, and quantification

Harvested embryos/organs were fixed in 2% paraformaldehyde at 4 °C for appropriate times depending on sample size, washed with PBS, dehydrated in methanol series (25, 50, 75, and 100%) for 15 min/each step at room temperature (RT) with rotating, and incubated in Dent bleach solution (1:2 = distilled water:15% DMSO in methanol) overnight (O/N) at RT. After bleaching, samples were serially rehydrated in 50 and 25% methanol and PBS for 15 min each at RT with rotating and washed in 0.1% PBST × 100 (0.1% Triton X-100 in PBS) for 2 h at RT. Then, samples were incubated in blocking solution (0.1% PBST × 100 with 3% milk/5% normal serum) and in primary antibodies against CD31 (550274, BD Pharmingen), Lyve1 (11-034, AngioBio), Endomucin (Emcn, sc-65495, Santa Cruz Biotechnology), Ter119 (550565, BD Pharmingen), and Nrp2 (AF567, R&D Systems) O/N at 4 °C. After washing, samples were incubated with secondary antibodies O/N at 4 °C, washed in PBST, fixed in 4% paraformaldehyde, and analyzed under a confocal laser microscope (LSM700, Carl Zeiss). The maximum intensity projection of embryo and organ images was obtained using z-stack function. Alternatively, after the reaction with primary antibodies, samples were washed and incubated with biotin-conjugated secondary antibodies O/N at 4 °C, washed, incubated with the avidin–biotin complex (ABC, PK-6100, Vector Laboratories) solution O/N at 4 °C, and treated with 3,3′-diaminobenzidine (DAB) solution (SK-4100, Vector Laboratories) until brown color was developed.

For X-gal staining, embryos were incubated in fixative solution (1% formaldehyde, 0.2% glutaraldehyde, 2 mM MgCl_2_, 5 mM EGTA, and 0.02% NP-40 in PBS) for 10 min at RT, washed thoroughly with PBS, and stained in X-gal solution (5 mM K_4_Fe(CN)_6_, 5 mM K_3_Fe(CN)_6_·3H_2_O, 2 mM MgCl_2_, 0.01% Na-deoxycholate, 0.02% NP-40, and 0.75 mg/ml X-gal in 100 mM phosphate buffer) O/N at 37 °C. Images of DAB- or X-gal-stained embryos/tissues were acquired using an Olympus stereo microscope.

Whole small intestine (from jejunum to ileum) was used to measure Lyve1(+) lymphatic coverage in *Dot1l*^*Δ*EC^, *Dot1l*^ΔLEC^, *Dot1l*^iΔLEC^, *Dot1l*^ΔdHSC^, *Dot1l*^iKO^, *Dot1l*^ΔHE^, m*Dot1l*^LECOE^, and m*Dot1l*^ECOE^ mesenteries. Then, the measurement of Lyve1(+) coverage of collecting lymphatics running parallel to Emcn(+) blood vessels was categorized into absent (no lymphatics), ≥50% (animals with more than half of lymphatics throughout mesenteries examined), <50% (animals with less than half of lymphatics throughout mesenteries examined), and complete (continuous lymphatics) as described in a study^4^. For vessel morphometric analyses in embryonic heads, diaphragm, heart, and skin, anatomically matched areas from experimental and control groups were chosen, and Lyve1(+) or CD31(+) vessel-branching points or lengths were measured using Zen (Carl Zeiss) and ImageJ software. To quantify Lyve1(+) areas of m*Dot1l*^ECOE^ and m*Dot1l*^LECOE^ mesenteries, pixel values of Lyve1(+) collecting lymphatics were measured as instructed by image quantification protocol of ImageJ software.

### Immunohistochemistry

Harvested embryos were fixed in 2% PFA solution O/N at 4 °C, washed with PBS, serially dehydrated in 50, 70, 95, and 100% ethanol for 30 min/each step at RT, incubated in xylene for 30 min, embedded in paraffin block, and cut into 7-µm sections. The sections were deparaffinized in xylene for 10 min, serially rehydrated in 100, 95, 70% ethanol, and PBS for 10 min/each step, and incubated with anti-Lyve1 and anti-Emcn antibodies (1:200 each) for 1 h at RT. After washing with PBS, slides were incubated with secondary antibodies for 1 h at RT, and colors (brown for Emcn and red for Lyve1) were developed using Polink DS-RRt-Hu/Ms A kit (DS211A-18, GBI Lab).

### Quantitative RT-PCR (qRT-PCR)

Total RNAs were extracted from cultured BECs or embryonic LECs using RNeasy Plus Mini Kit (74104, Qiagen), and cDNA was synthesized using SMARTer Pico PCR cDNA synthesis kit (634928, Takara) along with Advantage 2 PCR Kit (639206, Takara) according to the manufacturer’s instruction. qRT-PCR was performed in a StepOnePlus™ Systems (Applied Biosystems) using Fast SYBR® Green Master Mix (4385616, Applied Biosystems).

### Cell culture and magnetic-activated cell sorting (MACS)

Primary mouse dermal LECs derived from C57BL/6 embryos were obtained and maintained in complete mouse endothelial cell media with supplements (C57-6064L & M1168, Cell Biologics). All the in vitro cell culture experiments were performed within passage 5. For Dot1l inactivation, LECs were grown in the LEC culture media containing 2 µM EPZ5676 (reconstituted in DMSO, A12735, Adooq) for 7 days. The EPZ5676-treated LECs were subjected to ChIP-Seq analysis. Isolation of LECs from embryonic skin was described in a previous study^65^. Briefly, E15.5 embryonic skin was removed and enzymatically dissociated with media containing type II and IV collagenase, and DNaseI (LS004176, LS004188, and LS006344, respectively; Worthington Biochemical Corp.) for 20 min at 37 °C. After filtration through a 40-µm cell strainer, dissociated cells were incubated in both F4/80 and CD45 antibodies (13-4801 and 13-0451, respectively; eBioscience) for 1 h at RT to deplete macrophage and collected using goat anti-rat IgG-coated microbeads (130-048-101, Miltenyi Biotec). The F4/80(–)/CD45(–) cells were incubated with Lyve1 antibody (13-0443, eBioscience) and secondary antibodies. The Lyve1(+) LECs were collected and analyzed by RNA-Seq and qRT-PCR analyses.

### Lentivirus production and cell transduction

Catalytically dead Cas9 (dCas9) with guide RNAs (gRNAs) was used to overexpress *Dot1l* in BECs. Predicted gRNA sequences targeting *Dot1l* promoter or 5′ *Dot1l* upstream were obtained using CRISPR-ERA and Quilt tools. The designed gRNA sequences are as follows: *Dot1l*-OE1; 5′-TTGTTTGGCGTAAGTGCGTGCGTCGGT-3′, 5′-AAACACCGACGCACGCACTTACGCCAA-3′, *Dot1l*-OE2; 5′-CACCGTTTCCCCGGGTCCCCGCTTC-3′, 5′-AAACGAAGCGGGGACCCGGGGAAAC-3′, *Dot1l*-OE3; 5′-TCCCAGATTTGAACTTGACCCCGCC-3′, 5′-AAACGGCGGGGTCAAGTTCAAATCT-3′, *Dot1l*-OE4; 5′-CCTCGCGGAGGAGGGCGAGTCCAAG-3′, 5′-AAACCTTGGACTCGCCCTCCTCCGC-3′. After synthesis of gRNAs containing *Bbs*I sites, four candidate gRNAs were cloned into *Bbs*I-digested gRNA cloning vectors (Addgene # 53186, 53187, 53188, and 53189), and subjected to sequencing. Then, the four gRNAs and their promoters were subcloned into dCas9-containing lentivirus vector (Addgene # 59791) using golden gate method. Lenti-*Dot1l*^OE^ viruses were produced as described previously^66^. Briefly, HEK293T cells were grown in DMEM supplemented with 10% FBS and 1% Pen/Strep. Once cells reached ~85% confluency, lenti-*Dot1l*^OE^ and packaging vectors [psPAX2 (Addgene # 12260) and pMD2.G (Addgene # 12259) vectors] were transfected using Superfect reagent (Qiagen), and cells were maintained in Freestyle 293T media. Supernatant containing viral particle was harvested at 26, 38, and 50 h post transfection, and concentrated using Amicon Ultracell 100 K column (Amicon). The concentrated lentiviruses were transduced into BECs. Briefly, the cells were maintained in endothelial cell media, and transduced when cells reached ~50% confluency by using polybrene (10 µg/ml). After viral transduction, cells were fed with endothelial cell media supplemented with VEGF-C (100 ng/ml). Lenti-empty viruses were used as control. At 7 days post transduction, EGFP(+) cells were sorted using FACSAria (BD Biosciences) and used for qRT-PCR analysis.

### RNA-Seq and analysis

RNA-Seq experiments with pooled RNA samples extracted from 2 to 3 biological replicates were performed. Total RNA was extracted from control and *Dot1l*^*Δ*EC^ skin LECs using RNeasy Plus Mini Kit (74134, Qiagen), and its amount and quality of the total RNA were evaluated using Bioanalyzer (Agilent). RNA samples with >7.0 RNA Integrity Number (RIN) value were used for RNA-Seq library preparation with the ScriptSeq v2 kit (Illumina) according to the manufacturer’s instruction. Paired-end sequencing was performed on a MiSeq (Illumina), and reads were mapped to mm9 mouse genome using STAR tool (v2.5.2b, https://github.com/alexdobin/STAR)^67^. After mapping, fragments per kilobase million (FPKM) were calculated by Cufflinks (v2.2.1)^68^ tool using the following strand-specific Cuffnorm option: —library-type = fr-second strand. Functional annotation of differentially expressed genes (DEGs) and enrichment analyses were performed using DAVID (v6.8) and Gene Set Enrichment Analysis (GSEA, v2.2.4)^69^, respectively, and genes were considered differentially expressed at the fold change >3 and FPKM >5. R (v3.3.2) package was used for statistical analyses and scatter plot generation, and RNA-Seq results were visualized using Integrative Genomics Viewer (IGV)^70^.

### ChIP-Seq and analysis

ChIP-Seq experiments with pooled three biological replicates/group were performed. DMSO or EPZ5676-treated LECs were cross-linked with 1% formaldehyde (F8775, Sigma) for 10 min and neutralized with 0.125 M glycine (1610718, Bio-Rad). After washing with ice-cold PBS, cells were collected in a 1.5-ml tube and incubated in lysis buffer (5 mM PIPES, pH 8.0, 85 mM KCl, 1% NP-40, 1 mM PMSF, and 1× Protease inhibitor cocktail [11836153001, Roche]) for 15 min at 4 °C. After centrifugation, cell pellets were resuspended in 400 µl of nuclei lysis buffer (50 mM Tris-Cl, pH 8.0, 10 mM EDTA, pH 8.0, 1% SDS, 1 mM PMSF, and 1× Protease inhibitor cocktail) and incubated for 30 min at 4 °C. Nuclei were sonicated (Q500, Qsonica) for 20 cycles (30 s on/30 s off at 40% amplitude) at 4 °C to shear DNA into 300–400-bp fragments. After centrifugation, 2 ml of ice-cold IP dilution buffer (50 mM Tris-Cl, pH 7.5, 150 mM NaCl, 0.25% sodium deoxycholate, 1 mM EDTA, pH 8.0, 1% NP-40, 1 mM PMSF, and 1× Protease inhibitor cocktail) was added to the supernatant, and the sheared chromatin was incubated with the complex of H3K79me2 antibodies (ab3594, Abcam) and Dynabead-conjugated secondary antibodies (10004D, Life Technologies) O/N at 4 °C; a portion of non-immunoprecipitated chromatin was saved for input control. After washing, the immunoprecipitated DNA was treated with proteinase K (P2308, Sigma), extracted with phenol/chloroform, and precipitated with ethanol. DNA was dissolved in elution buffer (10 mM Tris-Cl, 5 mM EDTA, 300 mM NaCl, 0.5% SDS, and 2.5 μg/ml DNase-free RNase (11119915001, Roche)), and its amount and quality were evaluated using Bioanalyzer. ChIP-Seq libraries were produced using Truseq ChIP Sample kit (Illumina) according to the manufacturer’s instruction, and raw reads were aligned to mouse mm9 genome using Bowtie2 (v2.2.9); then, SAMtools (v1.2)^71^ was used for the data arrangement. ChIP-Seq peaks were called using the following MACS2 (v2.1.0)^72^ parameters: -B --nomodel -f BAM -g mm --broad -p 1e-5. NGS plot (v2.61) and seqMINER (v1.3.3e)^73^ were used for plotting read mean density and constructing a heat map, respectively. ChIP-Seq reads were visualized using the IGV. Disease-related genes, which showed downregulation of both gene expression and H3K79me2 enrichment by Dot1l inactivation, were identified using Ingenuity Pathway Analysis (IPA®, Qiagen).

### Statistical analysis

The data were analyzed by normality (Shapiro–Wilk test) and equal variance tests. Statistically significant differences in the continuous data of Lyve1(+) areas, vessel-branching points, and length of lymphatic vessels between groups were determined by a two-tailed *t* test using GraphPad Prism 5 (v5.01, GraphPad Software). The results were expressed as the mean ± s.e.m., and *p*-values less than 0.05 were considered significant.

## Supplementary information

Supplementary figure legend

Supplementary Fig. 1

Supplementary Fig. 2

Supplementary Fig. 3

Supplementary Fig. 4

Supplementary Table. 1

Supplementary Table. 2
