# Supplementary material for: Epigenetic priming by Dot1l in lymphatic endothelial progenitors ensures normal lymphatic development and function
Source: Cell Death Dis. 2020 Jan 6;11(1):14. doi: 10.1038/s41419-019-2201-1 (PMC6944698; doi:10.1038/s41419-019-2201-1)
Supplement: Supplementary file 1 — Supplementary figure legend [file 41419_2019_2201_MOESM1_ESM.pdf]

Supplementary Figure S1. Constitutive *Dot1l* KO causes underdevelopment of embryonic blood vessels whereas *Dot1l*$^{\Delta EC}$ embryos exhibit normal blood vessel development. a Representative images of whole-mount X-gal staining of E9.5-10.5 *Dot1l*$^{-/-}$ and littermate control embryos. Scale bar of E9.5=200µm, scale bar of E10.5=1mm . **b** Representative images of histology of E10.5 brain. Note that *Dot1l* KO causes dilation and reduces branching of brain blood vessels compared to control, and is lethal around E10.5. Red: nuclear fast red. Scale bar=1mm. **c** Representative images of whole-mount CD31 staining in E9.5 *Dot1l*$^{-/-}$ and control embryos. Scale bar=200µm. **d** Quantification of vessel branching points in E9.5 embryonic brains. n=5/group. Error bars show mean ± s.e.m. **e** Representative images of whole-mount Emcn immunofluorescent staining in the head of E10.5 *Dot1l*$^{\Delta EC}$ and control embryos. Scale bar=200µm. **f** Quantification of Emcn(+) vessel branching points in the head of E10.5 *Dot1l*$^{\Delta EC}$ (n=4) and control (n=5) embryos. NS; Not statistically significant. Error bars show mean ± s.e.m. **g** Representative images of whole-mount CD31 immunofluorescent staining in the skin of E17.5 *Dot1l*$^{\Delta EC}$ and control embryos. Scale bar=200µm.

Supplementary Figure S2. Lymphatic vessel defects in multiple organs of *Dot1l*$^{\Delta EC}$ embryos. a Representative bright field images of the E15.5 sternum. Scale bar=1 mm. **b**, **c**, **e**, **g** Representative whole-mount immunofluorescence images of Emcn, CD31, Lyve1 and Nrp2 in the E15.5 sternum (b, scale bar=200 µm), E17.5 diaphragm (c, scale bar=200 µm), E17.5 heart (e, scale bar=200 µm), and E14.5 skin (g, scale bar=2 mm (upper panel) and 500µm (lower panel)) of *Dot1l*$^{\Delta EC}$ embryos. White boxes in (b); E15.5 blood-filled sternum images presented in Fig. 1d are indicated. **d**, **f**, **h** Quantification of lymphatic vessel length (d and f) and branching points (h) in the organs. The numbers of control and *Dot1l*$^{\Delta EC}$ embryos analyzed were 5 and 3, 8 and 5, and 4 and 6, for the diaphragm, heart, and skin, respectively. *p<0.05 and ***p<0.001. Error bars show mean ± s.e.m.

Supplementary Figure S3. Cre activities in *Lyve1* $^{EGFP/Cre}$ strain. a Whole-mount X-gal staining in E10.5 *Lyve1*$^{EGFP/Cre}$;*R26R* embryo (scale bar=500μm) and immunohistochemistry (scale bar=100μm) with anti-Emcn antibody in section of the X-gal stained E10.5 embryo. Blue: X-gal. Brown: Emcn. **b** Whole-mount X-gal and Lyve1 staining in E17.5 mesentery. Blue: X-gal. Brown: Lyve1. Scale bar=200μm.

Supplementary Figure S4. Generation of mouse strain for targeted overexpression of mouse *Dot1l* in ECs and forced overexpression of *Dot1l* in mouse BECs. a Gene targeting strategy for conditional mouse *Dot1l* overexpression allele from *ROSA26* locus by homologous recombination. The targeting construct was designed to overexpress m*Dot1l-IRES-EGFP* under the control of *CMV* early enhancer/chicken β-actin (*CAG*) promoter after excision of 3X poly A by Cre recombinase. DTA; *Diphtheria toxin* A. tpA; three SV40 poly A signals. **b** Southern blot analysis after *Eco*RI digestion. Wildtype allele: 15.6kb, knock-in allele: 6.8kb. **c** Bright field and *Tie2*-Cre-mediated *EGFP* expression images of E17.5 *Dot1l*^{ECOE} mesentery. Scale bar=200μm. **d** EGFP expression in lenti-Dot1lOE-transduced BECs at post-transduction day 7 and FACS analysis. Scale bar=200μm.
