# Supplementary figures and images for: Epigenetic priming by Dot1l in lymphatic endothelial progenitors ensures normal lymphatic development and function
Source: Cell Death Dis. 2020 Jan 6;11(1):14. doi: 10.1038/s41419-019-2201-1 (PMC6944698; doi:10.1038/s41419-019-2201-1)

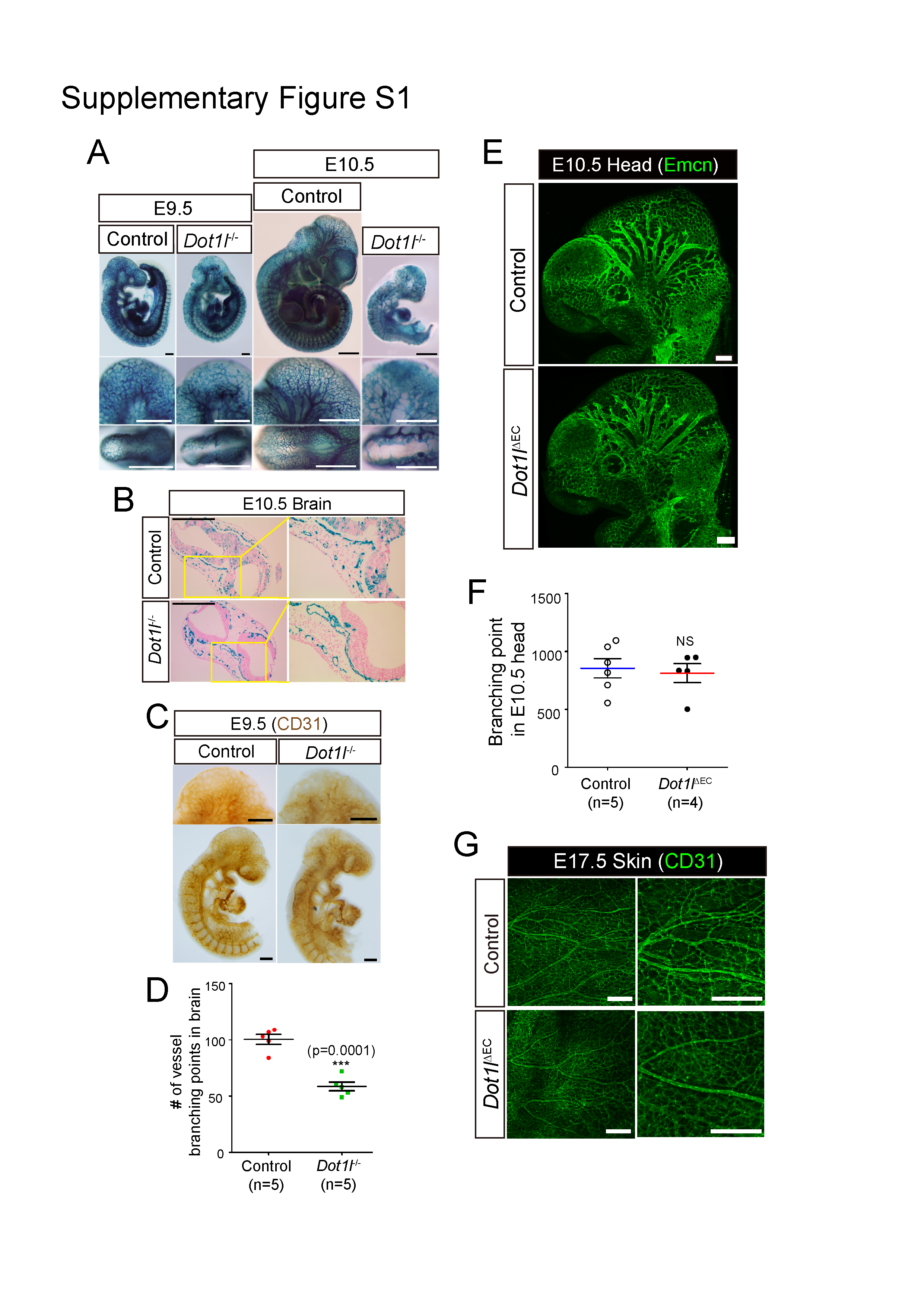

Supplement: Supplementary file 2 — Supplementary Fig. 1 [file 41419_2019_2201_MOESM2_ESM.tif]

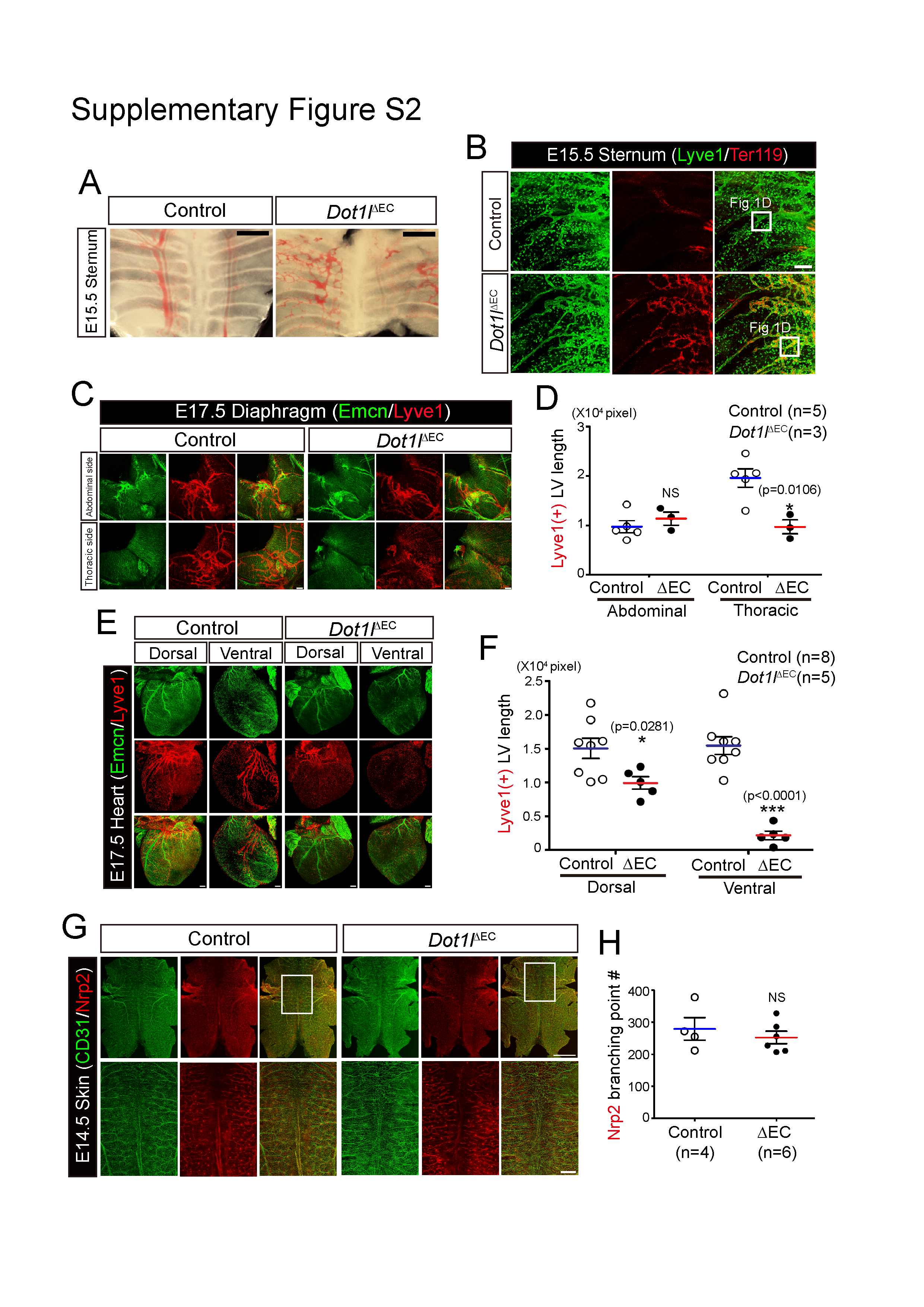

Supplement: Supplementary file 3 — Supplementary Fig. 2 [file 41419_2019_2201_MOESM3_ESM.tif]

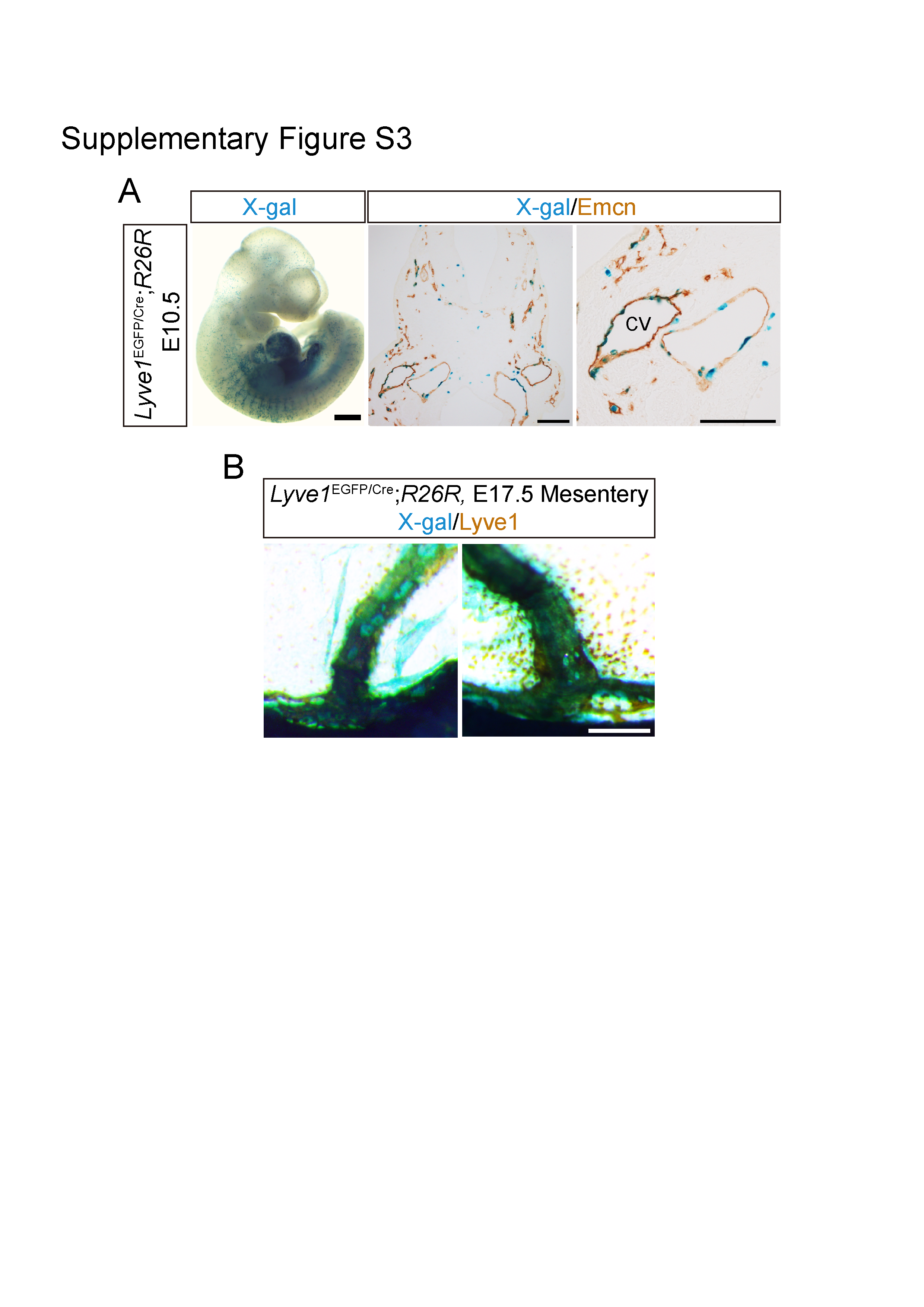

Supplement: Supplementary file 4 — Supplementary Fig. 3 [file 41419_2019_2201_MOESM4_ESM.tif]

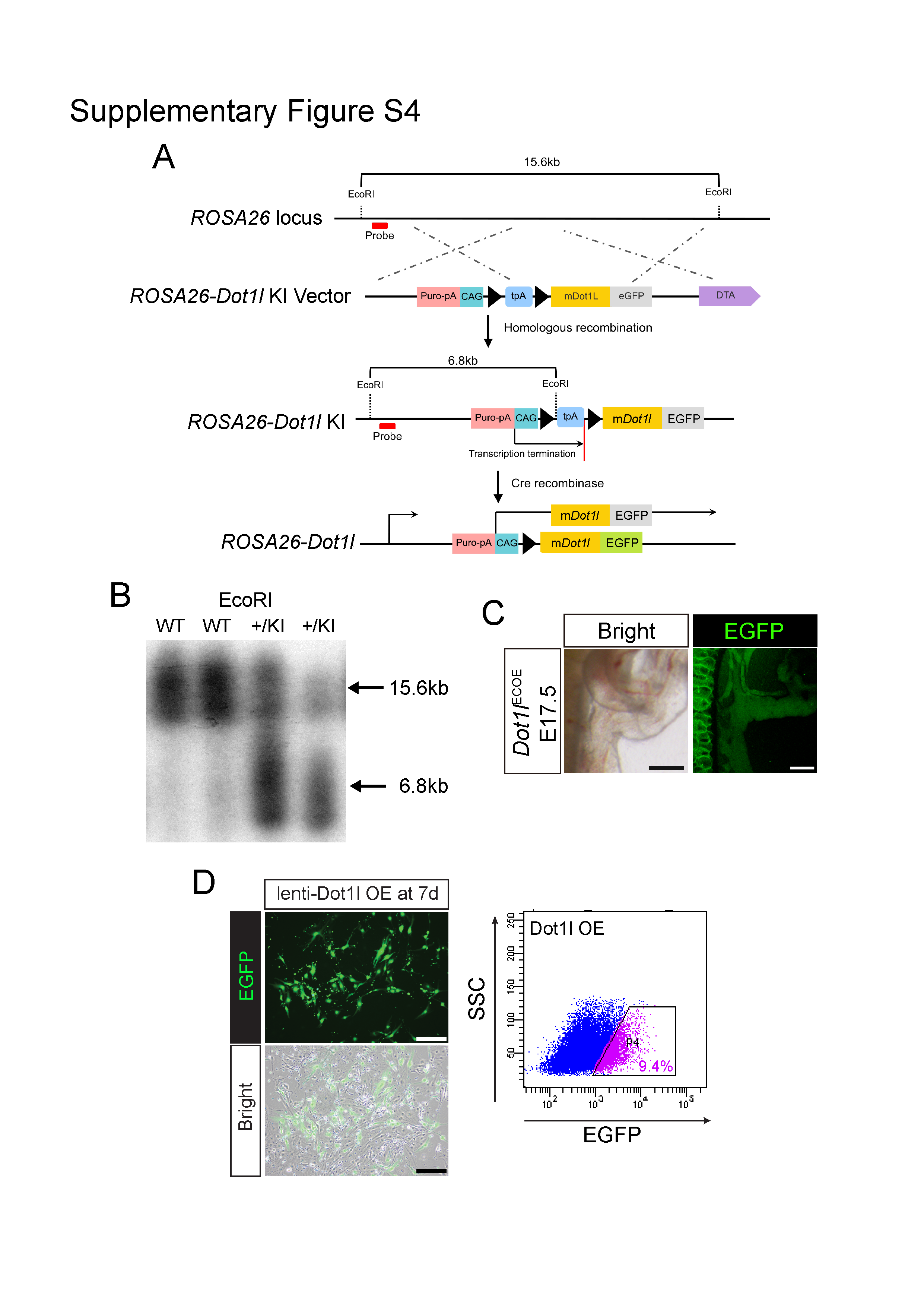

Supplement: Supplementary file 5 — Supplementary Fig. 4 [file 41419_2019_2201_MOESM5_ESM.tif]

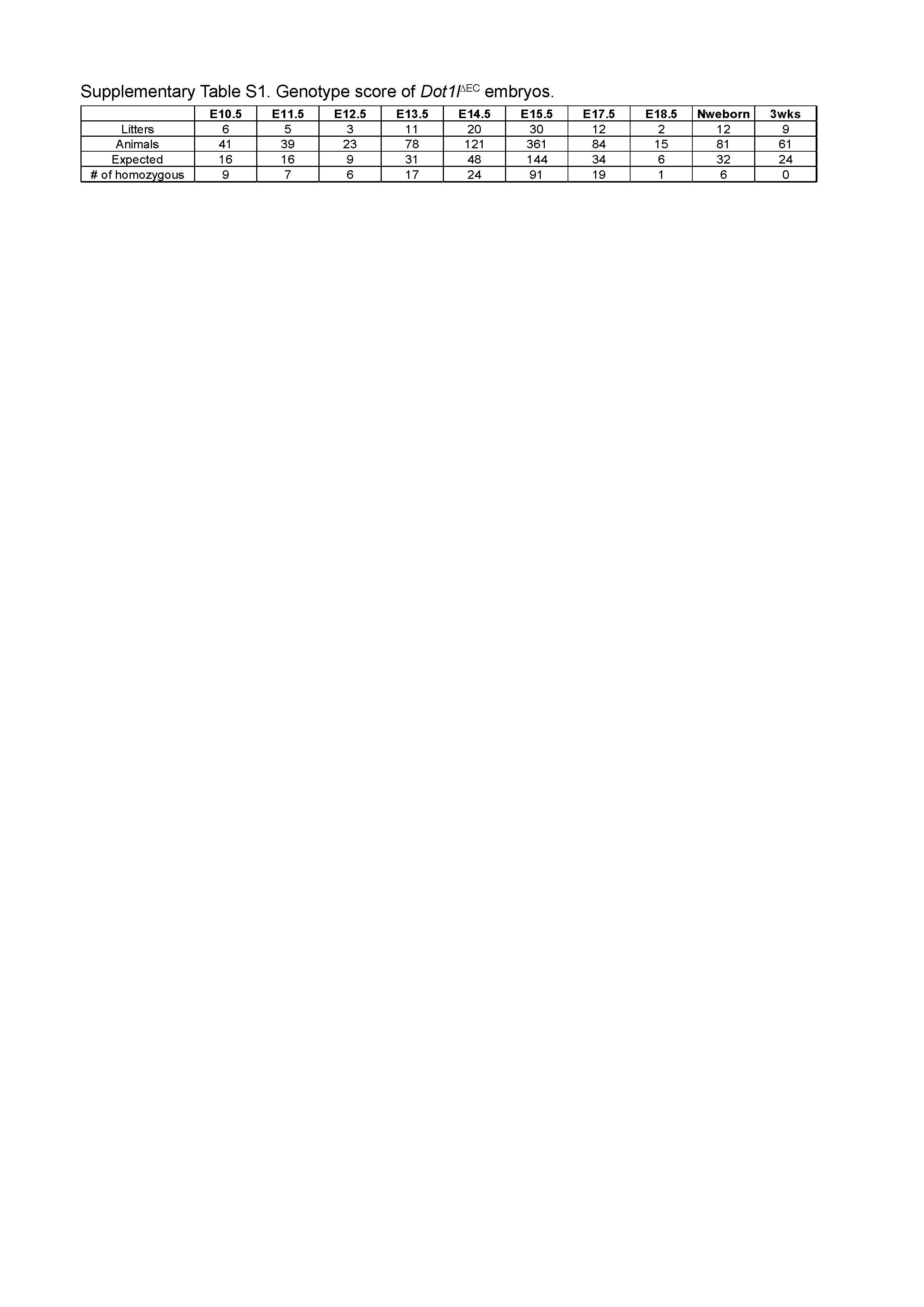

Supplement: Supplementary file 6 — Supplementary Table. 1 [file 41419_2019_2201_MOESM6_ESM.tif]

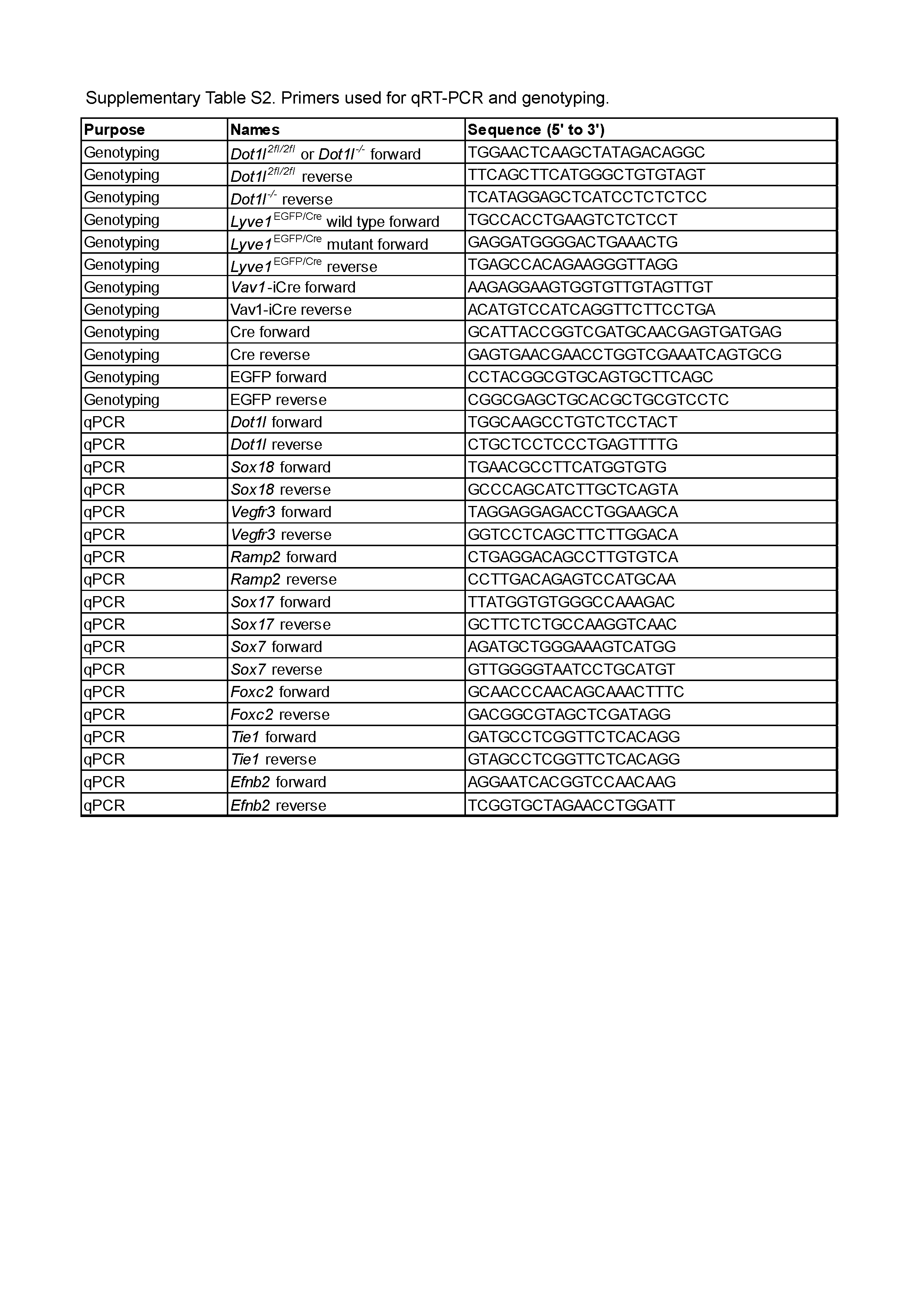

Supplement: Supplementary file 7 — Supplementary Table. 2 [file 41419_2019_2201_MOESM7_ESM.tif]
